# The involvement of effector memory CD4^+^ T cells in mediating the impact of genus *Oscillibacter* gut microbiota on Alzheimer’s disease: a Mendelian randomization study

**DOI:** 10.3389/fnagi.2024.1423707

**Published:** 2024-08-07

**Authors:** Huachang Zhang, Yudong Wang, Hui Zhao, Wei Wang, Fabin Han

**Affiliations:** ^1^The Institute for Tissue Engineering and Regenerative Medicine, Stem Cell and Regenerative Medicine Laboratory, Liaocheng People's Hospital/Liaocheng University, Liaocheng, Shandong, China; ^2^Department of Nursing, Liaocheng People’s Hospital, Liaocheng, Shandong, China; ^3^Intensive Care Unit, Liaocheng People’s Hospital, Liaocheng, Shandong, China; ^4^Henan Academy of Sciences, Zhengzhou, Henan, China; ^5^Henan High Tech Industrial Co., Ltd., Zhengzhou, Henan, China

**Keywords:** Mendelian randomization analysis, gut microbiota, Alzheimer’s disease, immune cells, GWAS

## Abstract

**Objective:**

This study aimed to investigate the causal relationship between gut microbiota characteristics (207 taxa and 205 pathways) and Alzheimer’s disease and determine and quantify the role of immune cells as potential mediators.

**Methods:**

Gut microbiota characteristics (207 taxa and 205 pathways) were obtained from the NHGRI-EBI GWAS Catalog project, while Alzheimer’s disease data and 731 immune cell characteristics were obtained from the IEU Open GWAS project. Two-sample Mendelian randomization (MR) was conducted to determine whether gut microbiota characteristics (207 taxa and 205 pathways) were causally related to Alzheimer’s disease. Furthermore, two-step MR was employed to quantify the proportion of the effect of immune cell characteristics mediated by gut microbiota characteristics (207 taxa and 205 pathways) on Alzheimer’s disease.

**Results:**

A total of 17 immune cell characteristics were identified as potential mediators for 13 gut microbiota influencing Alzheimer’s disease, with Effector Memory CD4+ T-cell Absolute Count accounted for 8.99% of the causal relationship between genus *Oscillibacter* and Alzheimer’s disease.

**Conclusion:**

In summary, our research confirms a causal relationship between gut microbiota and Alzheimer’s disease, with immune cells contributing to a significant portion of the effect. However, the full mediators of gut microbiota’s impact on Alzheimer’s disease remain unclear. Further investigation is warranted to explore additional potential risk factors acting as mediators.

## Introduction

Alzheimer’s disease (AD) is the most common cause of dementia worldwide, and its prevalence continues to grow (Weller and Budson, 2018; Scheltens et al., 2021). Current treatments, including cholinesterase inhibitors and memantine (Mossello and Ballini, 2012), improve symptoms but do not alter disease progression or life expectancy (Weller and Budson, 2018). Therefore, there continues to be an urgent need for the medical community to develop effective methods for early diagnosis and successful treatment of this progressive neurodegenerative disease.

There is mounting evidence suggesting that the microbiota may serve as a pivotal susceptibility factor in neurological disorders (Kau et al., 2011; le Chatelier et al., 2013; Sarkar et al., 2016; Bain and Cerovic, 2020). Studies have reported a notable decrease in anti-inflammatory bacteria, such as genera *Coprococcus*, *Roseburia*, and *Blautia*, in fecal samples of Parkinson’s disease (PD) patients (Keshavarzian et al., 2015), and a reduction in family *Bifidobacteriaceae* in AD patients (Vogt et al., 2017). In addition, studies have shown that microbes such as *Bacillus subtilis* can reduce α-synuclein levels by activating the DAF-2 signaling pathway directly and by indirectly modulating the sphingolipid metabolic pathway. Research using the 5XFAD mouse model of AD links microbial dysbiosis to activation of the endolysosomal CCAAT/enhancer-binding protein-β (C/EBP-β) and asparagine endopeptidase (AEP) pathway, leading to amyloid plaques and neurofibrillary tangles, indicating that gut microbiota can influence neuropathology (Padhi et al., 2022).

Recent research highlights the crucial interaction between gut microbiota and the host’s immune systems (Honda and Littman, 2016; Thaiss et al., 2016). Dysbiosis in gut microbiota can weaken immune responses and promote inflammatory diseases (Blander et al., 2017). For instance, lipopolysaccharide (LPS) produced by the gut microbiota has been detected in postmortem brain samples of AD patients (Calvani et al., 2018). In addition, peripheral LPS injection has been shown to induce microglial activation (DiCarlo et al., 2001), suggesting the involvement of the gut microbiota in regulating microglial activation and neuroinflammation in AD. In addition to resident immune cells in the brain, neuroinflammation associated with AD involves the infiltration of peripheral immune cells (Bryson and Lynch, 2016; Ajami et al., 2018; Mrdjen et al., 2018), including CD4^+^ and CD8^+^ T cells (Merlini et al., 2018). Wang et al. found that changes in the gut microbiota during AD progression lead to elevated levels of phenylalanine and isoleucine. These amino acids increased the proportion of Th1 cells in peripheral blood, promoting their infiltration into the brain. This leads to local crosstalk with M1 microglia, thereby inducing pathological neuroinflammation and cognitive impairment. These findings indicate that the gut microbiota and metabolites derived from amino acids can facilitate the infiltration of specific types of immune cells and drive neuroinflammation (Wang et al., 2019).

Mendelian Randomization (MR) analysis, which leverages genetic variations, is widely used to explore causal relationships between environmental exposures and diseases (Smith and Ebrahim, 2003). By using SNP-exposure and SNP-outcome associations from independent Genome-Wide Association Studies (GWAS), MR provides a unified causal estimate. With the rapid accumulation of GWAS data concerning gut microbiota, immune cells, and diseases (Orrù et al., 2020; Bellenguez et al., 2022; Lopera-Maya et al., 2022), it has become feasible to investigate large-scale interactions among these entities.

In this study, we used a two-step Mendelian Randomization approach to analyze the causal relationships between 412 gut microbiota species, 731 immune cells, and AD. Through this investigation, we aimed to elucidate the immunomodulatory mechanisms of gut microbiota and provide insights for the development of novel therapeutic approaches for AD patients.

## Methods

### Study design

**Figure 1:**
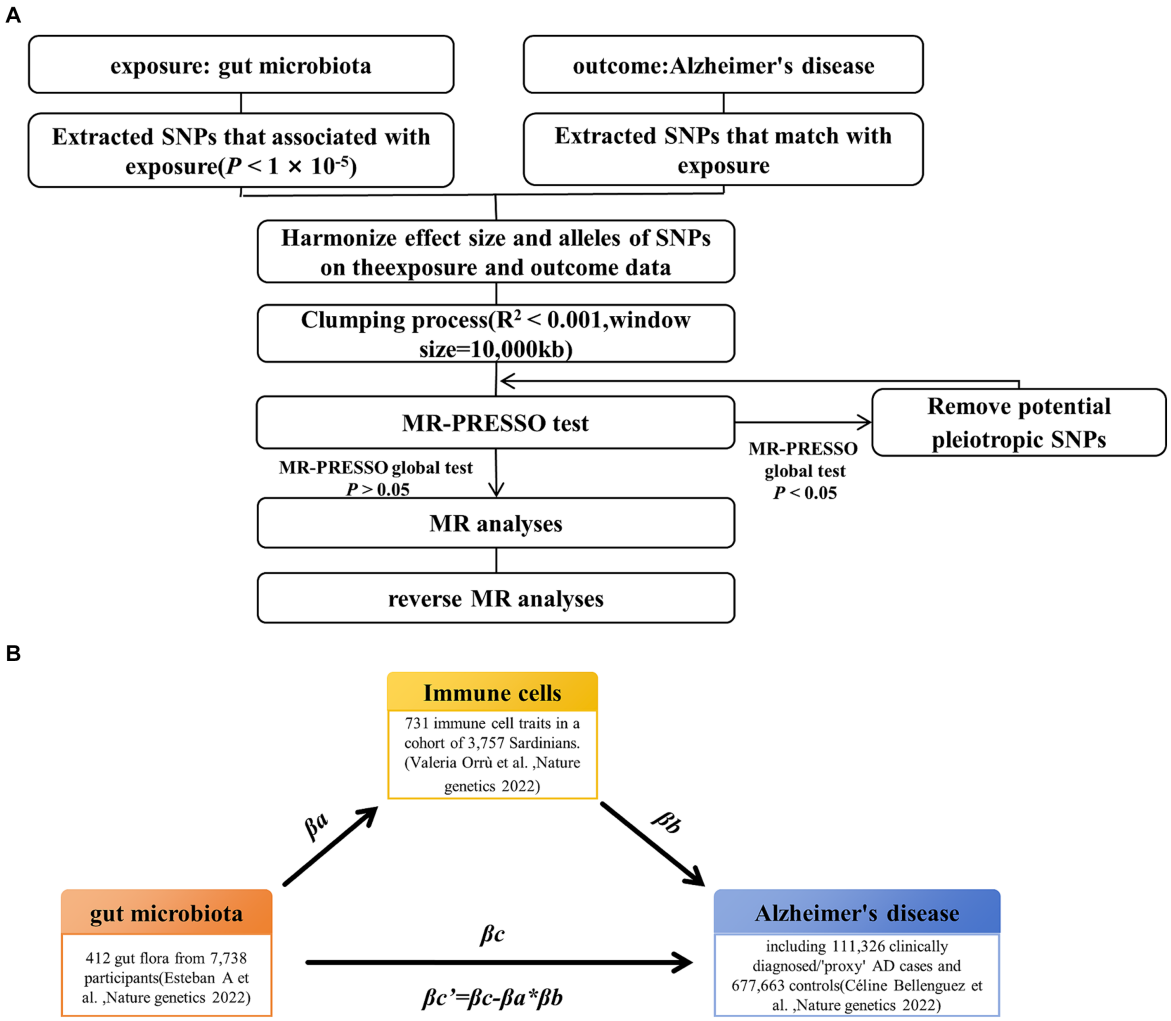
Study design and workflow. **(A)** Two-way Mendelian randomization flow chart. **(B)** Mediated Mendelian randomization. SNP, single nucleotide polymorphism; MR, Mendelian randomization.

The study’s workflow is illustrated in Figure 1. In essence, gut microbiota is regarded as the exposure, immune cell phenotypes serve as mediators, and AD is the outcome. Mendelian randomization and reverse Mendelian randomization analyses were conducted following instrumental variable selection. All statistical analyses were performed using R software packages, including two-sample MR and MR-PRESSO.

### Data of gut microbiota, Alzheimer’s disease, and immune cells

The data on gut microbiota composition and functionality were sourced from a study conducted by Lopera-Maya et al. (2022), encompassing 207 taxonomic groups and 205 pathways from 7,738 participants. This study identified 37 single nucleotide polymorphisms (SNPs) at 24 independent loci, meeting the genome-wide significance threshold of 5 × 10^−8^ for microbiota trait associations.

GWAS summary statistics for immune cell phenotypes, covering 118 absolute cell counts (e.g., T-cell absolute counts (AC): represents the absolute number of T cells), 389 median fluorescence intensities (e.g., CD4 on CD4+ T cell: represents the median fluorescence intensity of CD4 on CD4+ T cells), 32 morphological parameters (e.g., side scatter area (SSC-A) on B cell: represents the SSC-A of B cells), and 192 relative cell counts (e.g., T cell % leukocyte: represents the relative cell counts of T cells within leukocytes), were obtained from the IEU Open GWAS project database ^1^(Login numbers GCST90001391 to GCST90002121). This dataset reports the impact of approximately 22 million variants on 731 immune cell characteristics in a cohort of 3,757 Sardinian individuals. Notably, 122 significantly independent association signals (*p* < 1.28× 10^−11^) were detected for 459 cell traits at 70 loci, with 53 of these loci being newly identified (Orrù et al., 2020).

Summary statistics from GWAS for Alzheimer’s disease were obtained from the IEU Open GWAS project database (see text footnote 1). This analysis involved 487,511 individuals of European descent, including 39,106 cases and 46,828 controls. A total of 75 independent loci for AD were identified, comprising 33 previously reported and 42 newly discovered loci during the GWAS data analysis (Bellenguez et al., 2022). In addition, the two validation datasets were sourced from the IEU Open GWAS project database (see text footnote 1). Validation set one included 42,034 cases and 506,921 controls, and this study identified three new AD-related loci (*p* < 5 × 10^−8^) (Marioni et al., 2018). Validation set two included 53,042 cases and 355,900 controls, and this study used meta-analysis to identify 37 risk loci (Schwartzentruber et al., 2021).

### Instrumental variable selection

To ensure the reliability and accuracy of the conclusions regarding the causal relationship between gut microbiota and AD risk, as well as the immune cell characteristics as mediators, we implemented the following quality control steps to select the optimal instrumental variables (IVs) (Figure 1A). First, we selected significantly associated SNPs as IVs. For the gut microbiota and immune cells, we selected SNPs with a *p*-value below the genome-wide statistical significance threshold (1 × 10^−5^) as IVs. For AD, SNPs with a *p*-value below the genome-wide statistical significance threshold (5 × 10^−8^) were selected as IVs. Second, to mitigate potential bias resulting from strong linkage disequilibrium, we conducted a clumping process (*R*^2^ < 0.01 and clumping distance = 10,000 kb) to assess the linkage disequilibrium between the included SNPs. Third, we conducted weak instrumental variable selection based on *F*-values (*F* > 10). Fourth, we utilized MR-PRESSO to identify potential horizontal pleiotropy effects. The MR-PRESSO outlier test computed the *p*-value of significance for horizontal pleiotropy for each SNP, while the MR-PRESSO global test calculated the *p*-value of overall horizontal pleiotropy. If the *p*-value of the global test was greater than 0.05, MR analysis was conducted. Conversely, if the *p*-value was less than 0.05, SNPs were sorted in ascending order based on their MR-PRESSO outlier test *p*-values and then sequentially removed. After each SNP removal, the remaining SNPs underwent MR-PRESSO global testing. This recursive process continued until the *p*-value of the global test was not significant (*p* > 0.05). The remaining SNP list, after removing pleiotropic SNPs, was utilized for subsequent MR analysis.

### Primary analysis

Through two-sample MR analysis, we investigated the causal relationship between gut microbiota and AD and obtained the overall effect size βc. We utilized five prevalent MR methods to handle features containing multiple IVs: inverse variance-weighted (IVW) test (Burgess et al., 2013), weighted median estimator (WME) (Bowden et al., 2016), MR-Egger regression (Bowden et al., 2015), weighted mode (WM) (Hartwig et al., 2017), and MR-PRESSO (Verbanck et al., 2018). Research suggests that under specific conditions, the IVW method tends to offer more accurate results than others (Bowden et al., 2016); therefore, our findings primarily rely on IVW, with the other four methods serving as supplementary analyses. In addition, we employed Bayesian-weighted Mendelian randomization (BWMR) to validate the reliability of the results (Zhao et al., 2020). Subsequently, reverse Mendelian randomization was conducted with AD as the exposure and gut microbiota as the outcome to confirm the presence of the mediating effect.

### Mediation analysis

For the gut microbiota, which exist a mediating effect, we utilized a two-step MR approach to compute the total effect βa of gut microbiota on immune cells and the total effect βb of immune cells on AD. Subsequently, we derived the indirect effect of gut microbiota on AD as follows: βa * βb. The relationship between the direct effect and the total effect is expressed as βc’ = βc−βa * βb. In addition, using the delta method (Barton, 1998), we calculated the 95% confidence intervals. In cases where the directions of both the direct and indirect effects were the same, we also reported the proportion of the mediating effect, calculated as the indirect effect divided by the total effect (βa * βb / βc) (Figure 1B).

## Result

### Association of gut microbiota with AD

**Figure 2:**
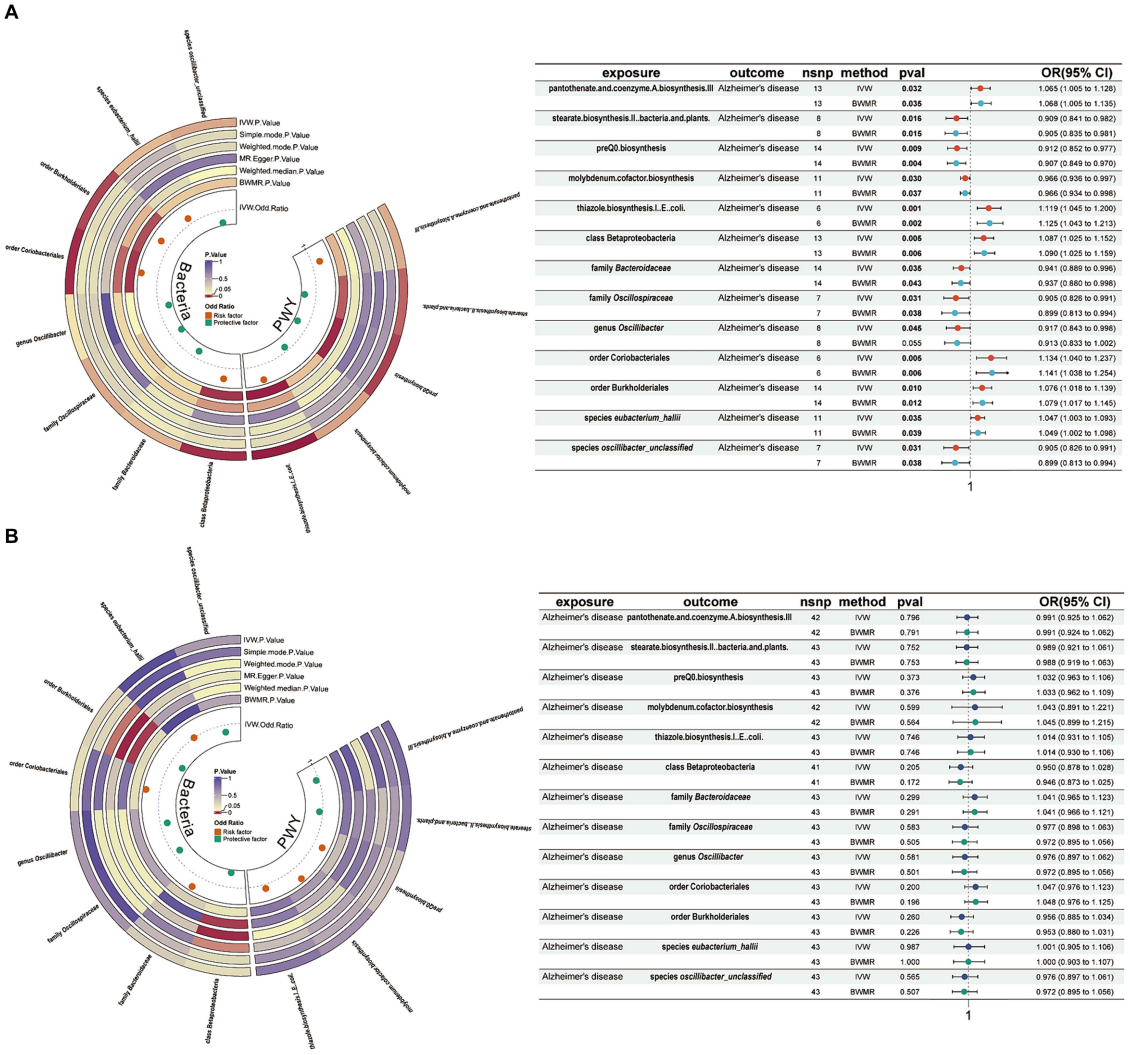
Mendelian randomization results of causal effects between gut microbiome and AD. **(A)** Gut microbiome as exposure and AD as outcome. **(B)** AD as exposure and gut microbiome as outcome. AD, Alzheimer’s disease; IVW, inverse variance-weighted; BWMR, Bayesian-weighted Mendelian randomization.

Using a threshold of 1 × 10^−5^, instrumental variables were selected for 412 gut microbiota features (207 taxa and 205 pathways) for bidirectional MR analysis. Among them, 5 gut microbiota pathways and 7 gut microbiota taxa were found to be associated with AD. Pantothenate and coenzyme A biosynthesis III (OR = 1.065, 95% CI = 1.005–1.128, *p* = 0.032, IVW), thiazole biosynthesis I in *E. coli* (OR = 1.119, 95% CI = 1.045–1.200, *p* = 0.001, IVW), class Betaproteobacteria (OR = 1.087, 95% CI = 1.005–1.128, *p* = 0.005, IVW), order Coriobacteriales (OR = 1.065, 95% CI = 1.025–1.152, *p* = 0.005, IVW), order Burkholderiales (OR = 1.076, 95% CI = 1.018–1.139, *p* = 0.010, IVW), and species *eubacterium_hallii* (OR = 1.047, 95% CI = 1.003–1.093, *p* = 0.035, IVW) were associated with an increased risk of AD. Conversely, stearate biosynthesis II in bacteria and plants (OR = 0.909, 95% CI = 0.841–0.982, *p* = 0.016, IVW), preQ0 biosynthesis (OR = 0.912, 95% CI = 0.852–0.977, *p* = 0.009, IVW), molybdenum cofactor biosynthesis (OR = 0.966, 95% CI = 0.936–0.997, *p* = 0.030, IVW), family *Bacteroidaceae* (OR = 0.941, 95% CI = 0.889–0.996, *p* = 0.035, IVW), family *Oscillospiraceae* (OR = 0.905, 95% CI = 0.826–0.991, *p* = 0.031, IVW), genus *Oscillibacter* (OR = 0.917, 95% CI = 0.843–0.998, *p* = 0.045, IVW), and species *oscillibacter_unclassified* (OR = 0.905, 95% CI = 0.826–0.991, *p* = 0.031, IVW) (Figure 2A) were associated with a decreased risk of AD. These results were validated using BWMR. Subsequently, reverse MR analysis was conducted to validate whether AD affects gut microbiota, and the results showed no effect of AD on the aforementioned 5 gut microbiota pathways and 7 gut microbiota taxa (Figure 2B).

### Association of gut microbiota with immune cells

**Figure 3:**
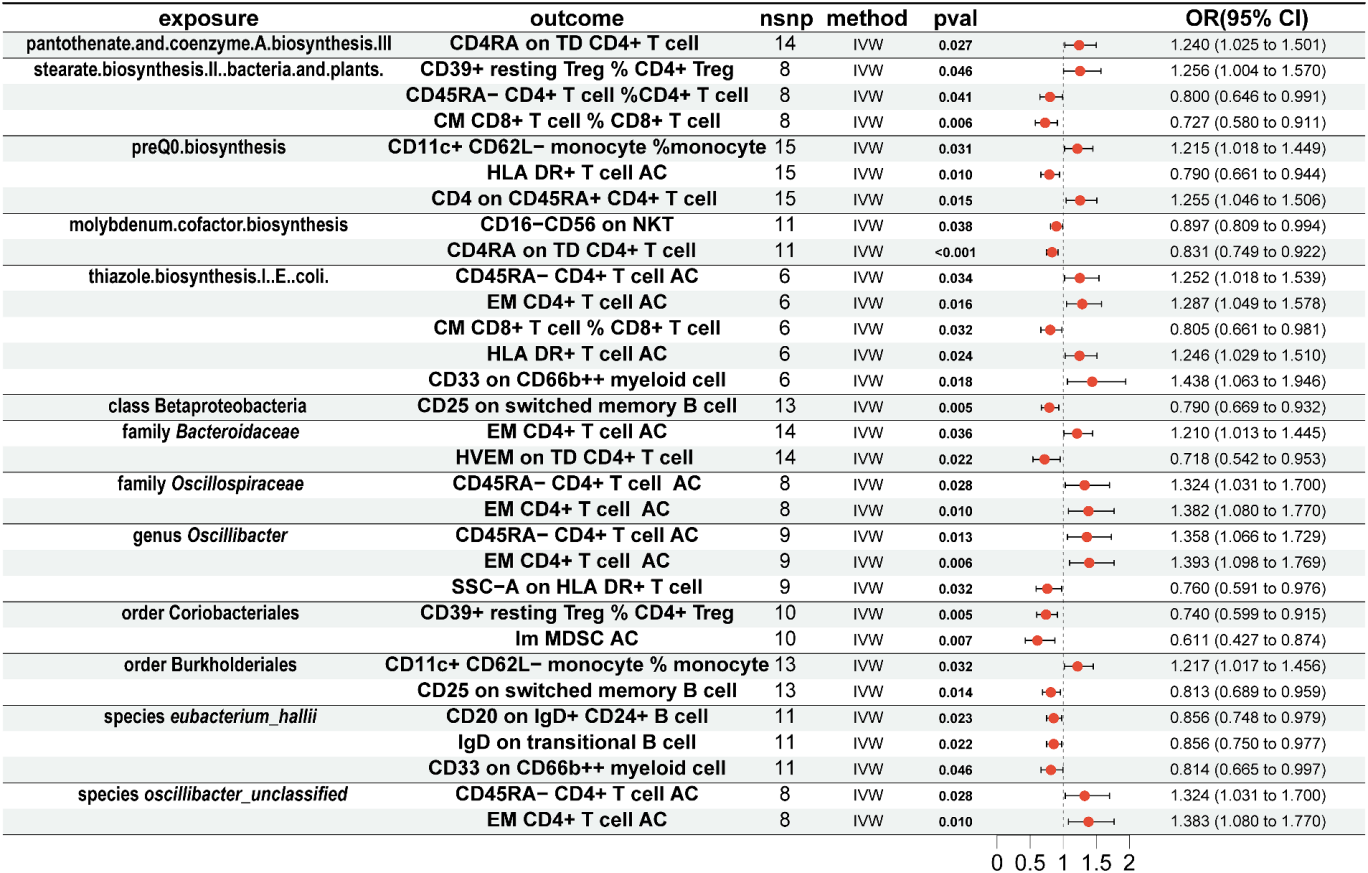
Mendelian randomization results of causal effects between gut microbiome and immune cells. Gut microbiome as exposure and immune cells as outcome.

To explore whether gut microbiota associated with AD affects immune cell phenotypes, we used a threshold of 1 × 10^−5^ to select the aforementioned 13 gut microbiota-related SNPs as instrumental variables for MR analysis with 731 immune cell phenotypes. The results showed that for 5 gut microbiota pathways, pantothenate and coenzyme A biosynthesis III were positively associated with CD4RA on terminally differentiated (TD) CD4^+^ T cell (OR = 1.240, 95% CI = 1.025–1.501, *p* = 0.027, IVW), and stearate biosynthesis II in bacteria and plants was positively associated with CD39^+^ resting CD4^+^ regulatory T cell (Treg) %CD4^+^ Treg (OR = 1.256, 95% CI = 1.004–1.570, *p* = 0.046, IVW) and negatively associated with CD45RA-CD4^+^ T cell % CD4^+^ T cell (OR = 0.800, 95% CI = 0.646–0.991, *p* = 0.041, IVW) and central memory (CM) CD8^+^ T cell % CD8^+^ T cell (OR = 0.727, 95% CI = 0.580–0.911, *p* = 0.727, IVW). PreQ0 biosynthesis was positively associated with CD11c^+^ CD62L-monocyte % monocyte (OR = 1.215, 95% CI = 1.018–1.449, *p* = 0.031, IVW) and CD4 on CD45RA^+^ CD4^+^ T cell (OR = 1.255, 95% CI = 1.046–1.506, *p* = 0.015, IVW) and negatively associated with HLA DR^+^ T-cell AC (OR = 0.790, 95% CI = 0.661–0.944, *p* = 0.010, IVW). Molybdenum cofactor biosynthesis was negatively associated with CD16-CD56 on natural killer T (NKT) (OR = 0.897, 95% CI = 0.809–0.994, *p* = 0.038, IVW) and CD4RA on TD CD4^+^ T cell (OR = 0.831, 95% CI = 0.749–0.922, *p* < 0.001, IVW). Thiazole biosynthesis I in *E. coli* was positively associated with CD45RA-CD4^+^ T-cell AC (OR = 1.252, 95% CI = 1.018–1.539, *p* = 0.034, IVW), effector memory (EM) CD4^+^ T-cell AC (OR = 1.287, 95% CI = 1.049–1.578, *p* = 0.016, IVW), HLA DR^+^ T-cell AC (OR = 1.246, 95% CI = 1.029–1.510, *p* = 0.024, IVW), and CD33 on CD66b^++^ myeloid cell (OR = 1.438, 95% CI = 1.063–1.946, *p* = 0.018, IVW) and negatively associated with CM CD8^+^ T cell % CD8^+^ T cell (OR = 0.805, 95% CI = 0.661–0.981, *p* = 0.032, IVW) (Figure 3).

For 7 gut microbiota taxa, class Betaproteobacteria was negatively associated with CD25 on switched memory B cell (OR = 0.790, 95% CI = 0.669–0.932, *p* = 0.005, IVW), and family *Bacteroidaceae* was positively associated with EM CD4^+^ T-cell AC (OR = 1.210, 95% CI = 1.013–1.445, *p* = 0.036, IVW) and negatively associated with herpes virus entry mediator (HVEM) on TD CD4^+^ T cell (OR = 0.718, 95% CI = 0.542–0.953, *p* = 0.022, IVW). Family *Oscillospiraceae* was positively associated with CD45RA-CD4^+^ T-cell AC (OR = 1.324, 95% CI = 1.031–1.700, *p* = 0.028, IVW) and EM CD4^+^ T-cell AC (OR = 1.382, 95% CI = 1.080–1.770, *p* = 0.010, IVW); genus *Oscillibacter* was positively associated with CD45RA-CD4^+^ T-cell AC (OR = 1.358, 95% CI = 1.066–1.729, *p* = 0.013, IVW) and EM CD4^+^ T-cell AC (OR = 1.393, 95% CI = 1.098–1.769, *p* = 0.006, IVW) and negatively associated with SSC-A on HLA DR^+^ T cell (OR = 0.760, 95% CI = 0.591–0.976, *p* = 0.032, IVW); order Coriobacteriales was negatively associated with CD39^+^ resting CD4^+^ Treg % CD4^+^ Treg (OR = 0.740, 95% CI = 0.599–0.915, *p* = 0.005, IVW) and immature myeloid-derived suppressor cells (Im MDSC) AC (OR = 0.611, 95% CI = 0.427–0.874, *p* = 0.007, IVW); order Burkholderiales was positively associated with CD11c^+^ CD62L-monocyte % monocyte (OR = 1.217, 95% CI = 1.017–1.456, *p* = 0.032, IVW) and negatively associated with CD25 on switched memory B cell (OR = 0.813, 95% CI = 0.689–0.959, *p* = 0.014, IVW); species *eubacterium_hallii* was negatively associated with CD20 on IgD^+^ CD24^+^ B cell (OR = 0.856, 95% CI = 0.748–0.979, *p* = 0.023, IVW), IgD on transitional B cell (OR = 0.856, 95% CI = 0.750–0.977, *p* = 0.022, IVW), and CD33 on CD66b^++^ myeloid cell (OR = 0.814, 95% CI = 0.665–0.997, *p* = 0.046, IVW); species *oscillibacter_unclassified* was positively associated with CD45RA-CD4^+^ T-cell AC (OR = 1.324, 95% CI = 1.031–1.700, *p* = 0.028, IVW) and EM CD4^+^ T-cell AC (OR = 1.383, 95% CI = 1.080–1.770, *p* = 0.010, IVW).

### Association of immune cells with AD

**Figure 4:**
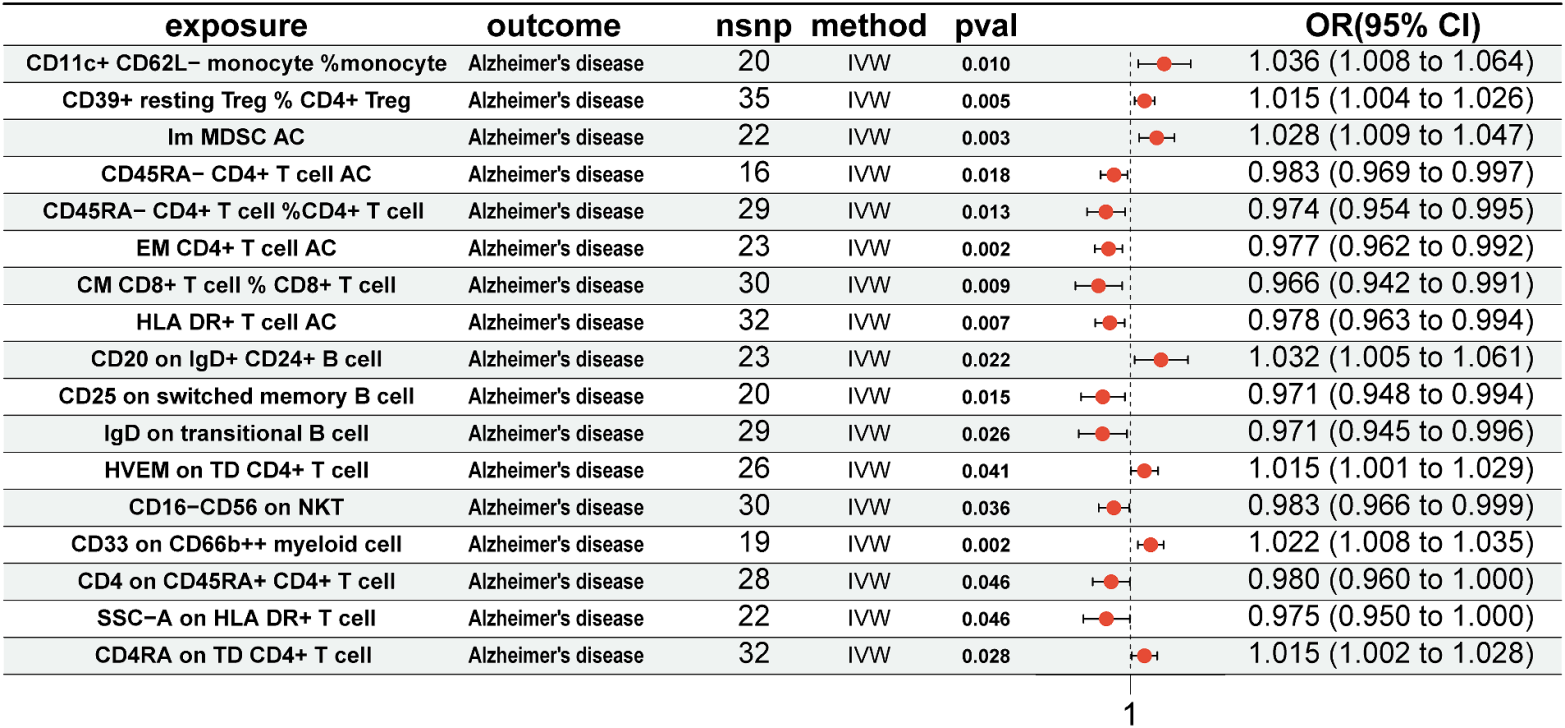
Mendelian randomization results of causal effects between immune cells and AD. Immune cells as exposure and AD as outcome. AD, Alzheimer’s disease.

For immune cells associated with the gut microbiota, we conducted MR analysis with AD. The results revealed that CD11c^+^ CD62L-monocyte % monocyte (OR = 1.036, 95% CI = 1.008–1.064, *p* = 0.010, IVW), CD39^+^ resting CD4^+^ Treg % CD4^+^ Treg (OR = 1.015, 95% CI = 1.004–1.026, *p* = 0.005, IVW), Im MDSC AC (OR = 1.028, 95% CI = 1.009–1.047, *p* = 0.003, IVW), CD20 on IgD^+^ CD24^+^ B cell (OR = 1.032, 95% CI = 1.005–1.061, *p* = 0.022, IVW), HVEM on TD CD4+ T cell (OR = 1.015, 95% CI = 1.001–1.029, *p* = 0.041, IVW), CD33 on CD66b^++^ myeloid cell (OR = 1.022, 95% CI = 1.008–1.035, *p* = 0.002, IVW), and CD4RA on TD CD4^+^ T cell (OR = 1.015, 95% CI = 1.002–1.028, *p* = 0.028, IVW) were positively associated with AD. Conversely, CD45RA-CD4^+^ T-cell AC (OR = 0.983, 95% CI = 0.969–0.997, *p* = 0.018, IVW), CD45RA-CD4^+^ T cell %CD4^+^ T cell (OR = 0.974, 95% CI = 0.954–0.995, *p* = 0.013, IVW), EM CD4^+^ T-cell AC (OR = 0.977, 95% CI = 0.962–0.992, *p* = 0.002, IVW), CM CD8^+^ T cell % CD8^+^ T cell (OR = 0.966, 95% CI = 0.942–0.991, *p* = 0.009, IVW), HLA DR^+^ T-cell AC (OR = 0.978, 95% CI = 0.963–0.994, *p* = 0.007, IVW), CD25 on switched memory B cell (OR = 0.971, 95% CI = 0.948–0.994, *p* = 0.015, IVW), IgD on transitional B cell (OR = 0.971, 95% CI = 0.945–0.996, *p* = 0.026, IVW), CD16-CD56 on NKT (OR = 0.983, 95% CI = 0.966–0.999, *p* = 0.036, IVW), CD4 on CD45RA^+^ CD4^+^ T cell (OR = 0.980, 95% CI = 0.960–1.000, *p* = 0.046, IVW), and SSC-A on HLA DR^+^ T cell (OR = 0.975, 95% CI = 0.950–1.000, *p* = 0.046, IVW) were negatively associated with AD (Figure 4).

### Association between immune cell-mediated gut microbiota and AD

**Figure 5:**
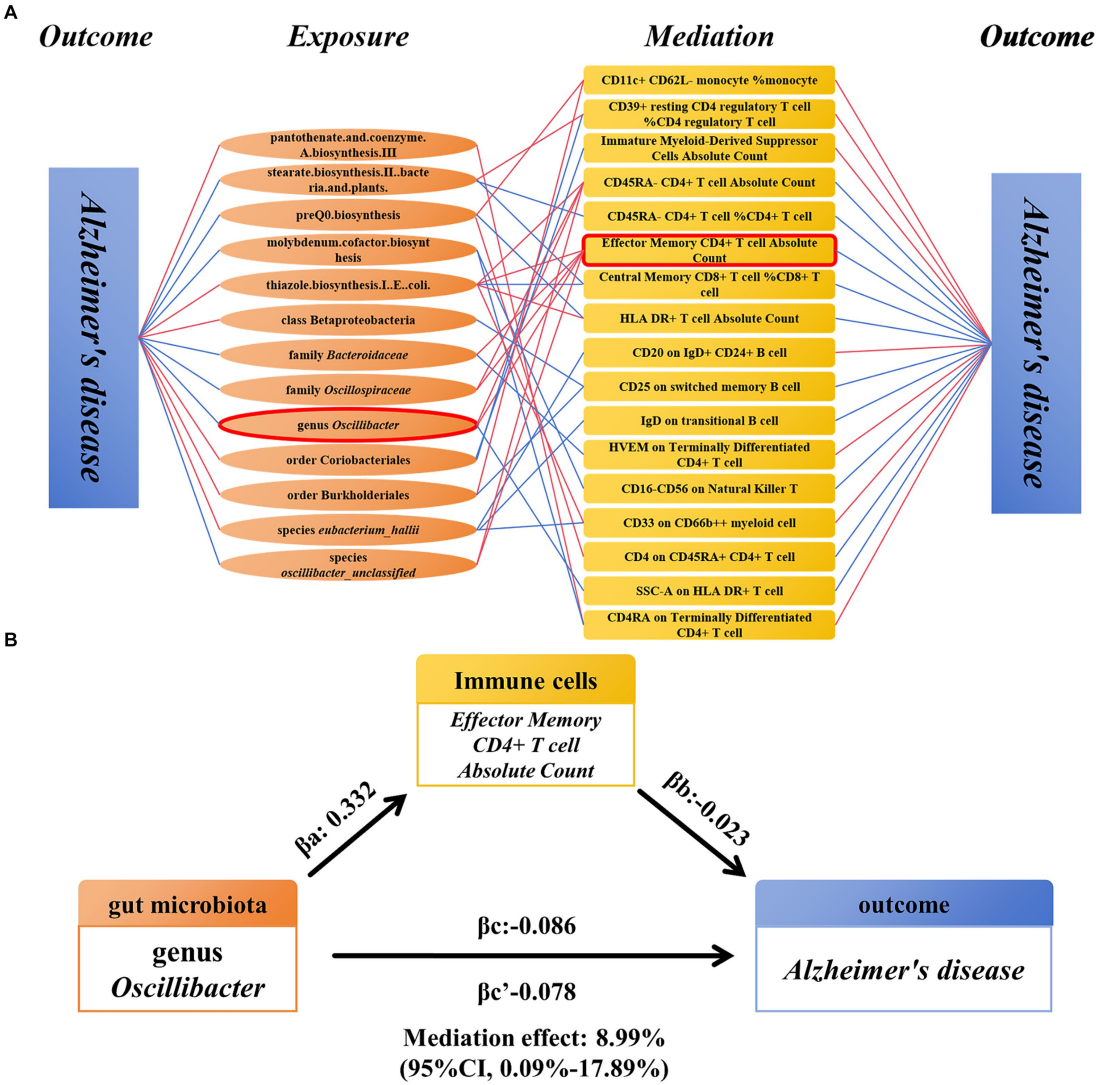
Mendelian randomization analysis of the relationship between immune cell-mediated gut microbiota and AD. **(A)** The relationship between the gut microbiota and AD mediated by 17 types of immune cells. **(B)** Effector Memory CD4^+^ T-cell Absolute Count mediated genus *Oscillibacter* and the relationship between AD. AD, Alzheimer’s disease.

We analyzed immune cells as mediators between gut microbiota and AD. The results indicate associations between 13 gut microbiota and 17 immune cells, which in turn are associated with AD (Figure 5A) (Supplementary Table S7). Among these, EM CD4^+^ T-cell AC showed significant mediation in the pathway from genus *Oscillibacter* to AD. In the risk reduction associated with genus *Oscillibacter*, EM CD4^+^ T cell AC accounted for 8.99% (proportion mediated: 8.99%; 95% CI = 0.09%–17.89%; *p* = 0.048) (Figure 5B).

### Utilize the AD validation set for conducting validation analysis

**Figure 6:**
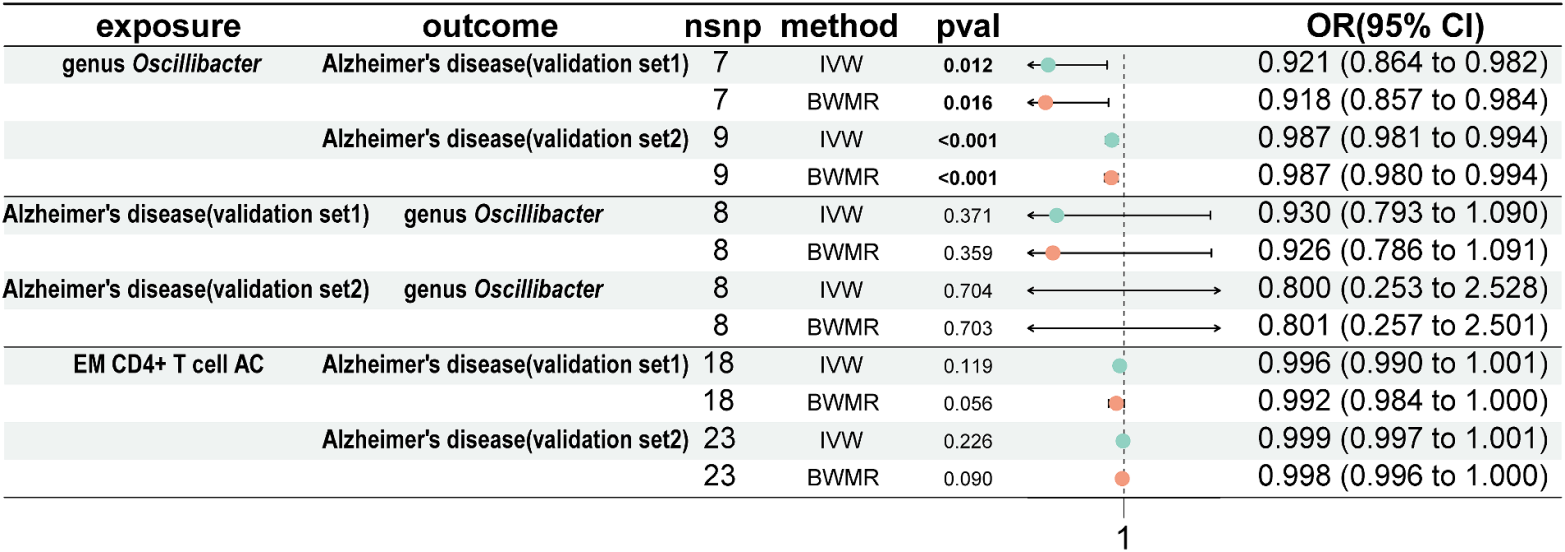
Analyze the validation sets using Mendelian randomization. Using genus *Oscillibacter*, AD, and EM CD4^+^ T-cell AC, respectively, as exposures corresponding to AD, with genus *Oscillibacter* and AD as outcomes. AD, Alzheimer’s disease; EM CD4^+^ T-cell AC, Effector Memory CD4^+^ T-cell Absolute Count.

To validate our results, we conducted validation using 2 AD GWAS datasets. The findings indicated that genus *Oscillibacter* was associated with a decreased risk of AD (AD validation set 1: OR = 0.921, 95% CI = 0.864–0.982, *p* = 0.012, IVW; AD validation set 2: OR = 0.987, 95% CI = 0.981–0.994, *p* < 0.001, IVW). Reverse MR analysis showed that AD had no impact on genus *Oscillibacter*. In addition, EM CD4^+^ T-cell AC had no effect on the 2 AD validation sets (Figure 6).

### Sensitivity analysis

Some sensitivity analyses were conducted to examine and correct for pleiotropy in causal estimates. In our previous analysis, when using the MR-Egger regression intercept method, evidence of horizontal pleiotropy was found only for the reverse MR of class Betaproteobacteria and order Burkholderiales on AD, while no evidence of horizontal pleiotropy was found for the remaining instrumental variables (*p* > 0.05). In addition, MR-PRESSO analysis revealed no outliers among the instrumental variables used in all analyses. Furthermore, Cochrane’s Q statistic showed no significant heterogeneity (*p* > 0.05) (Supplementary Tables S1–S6).

## Discussion

Many studies have indicated a link between alterations in gut microbiota composition and the progression of AD. To systematically elucidate beneficial and harmful microbial communities in the gut microbiota related to AD, we employed a two-sample MR approach for analysis. Our findings suggest that three gut microbiota pathways, namely, stearate biosynthesis II in bacteria and plants, preQ0 biosynthesis, and molybdenum cofactor biosynthesis, along with four microbial taxa family *Bacteroidaceae*, family *Oscillospiraceae*, genus *Oscillibacter*, and species *oscillibacter_unclassified* may serve as protective factors against AD. Previous research has suggested a decrease in phylum Firmicutes and an increase in phylum Bacteroidetes in AD transgenic mouse models (Harach et al., 2017; Zhang et al., 2017), implying that phylum Bacteroidetes may act as a risk factor for AD. However, our analysis revealing family *Bacteroidaceae* as a protective factor may be explained by potential masking effects from certain taxa within phylum Bacteroidetes. Furthermore, research conducted by Cattaneo et al. has indicated a reduction in the abundance of species *Bacteroides fragilis* in the fecal samples of AD patients (Cattaneo et al., 2017). In addition, studies have shown a significant decrease in phylum Bacteroidetes in mice induced with amyloid-beta (Aβ) (Xu et al., 2020), and other animal studies have reported a decrease in phylum Bacteroidetes (Park et al., 2017), which indirectly supports our analysis findings.

An increasing number of AD treatment studies are now observing changes in gut microbiota post-treatment. Wang et al. found that exercise can improve AD symptoms and gut microbiota in AD mice, with exercise increasing the abundance of genus *Oscillibacter* (Wang et al., 2021). Turmeric has been used to treat AD mice, and mice treated with turmeric showed recovery of some beneficial gut microbiota at the genus and species levels. Specifically, *Oscillospiraceae* and *Rikenellaceae* were enriched at the family level, while at the genus level, the abundance of *Oscillibacter*, *Alistipes*, *Pseudoflavonifractor*, *Duncaniella*, and *Flintibacter* increased (Lamichhane et al., 2024). These findings align with our analysis, which identified family *Oscillospiraceae* and genus *Oscillibacter* as protective factors against AD. Furthermore, our results indicate that at the next taxonomic level (species level) of genus *Oscillibacter*, species *oscillibacter_unclassified* also acts as a protective factor against AD. Conversely, two pathways pantothenate and coenzyme A biosynthesis III, and thiazole biosynthesis I in *E. coli* along with four gut microbiota taxa class Betaproteobacteria, order Coriobacteriales, order Burkholderiales, and species *eubacterium_hallii* may act as risk factors for AD. These pathways are critical for synthesizing CoA and thiamine and essential for energy metabolism, stress response, and enzymatic functions crucial to microbial growth and resilience (Begley et al., 1999; Chatterjee et al., 2007; Leonardi and Jackowski, 2007). Dysregulated metabolic pathways and microbial imbalances can disrupt these essential processes, potentially leading to metabolic deficiencies, reduced stress tolerance, and compromised cellular health. Such disruptions may exacerbate neuroinflammatory processes and compromise the gut–brain axis, highlighting their potential contribution to the development and progression of AD.

In addition to gut microbiota, the role of immune cells in AD has also been increasingly recognized. Studies by Hui Xu and Yanjun Lu′s teams have reported that the proportion of CD4^+^ T cells in the AD group is significantly higher than that in the normal control group, suggesting that CD4^+^ T-cell subsets may play a role in the pathogenesis of AD (Lu et al., 2021; Xu and Jia, 2021). However, these studies did not provide a more detailed subdivision of CD4^+^ T-cell subsets. Our research confirms and further refines these conclusions, finding potential causal associations between various CD4^+^ T-cell subsets and AD. Specifically, CD39^+^ resting CD4^+^ Treg % CD4^+^ Treg, HVEM on TD CD4^+^ T cell, and CD4RA on TD CD4^+^ T cell may act as harmful factors in AD, whereas CD45RA-CD4^+^ T-cell AC, CD45RA-CD4^+^ T cell % CD4^+^ T cell, EM CD4^+^ T-cell AC, CD25 on switched memory B cell, and CD4 on CD45RA^+^ CD4^+^ T cell may serve as protective factors. In addition, since increased brain CD33 expression is associated with higher amyloid plaque load, Gu et al. (2022) used a Mendelian randomization approach to show that CD33 levels on CD66b^++^ myeloid cells in peripheral blood are associated with a high risk of AD, a finding that our study also confirmed. In addition to these results, we also discovered several previously unreported immune cell phenotypes related to AD, including harmful factors such as CD11c^+^ CD62L-monocyte % monocyte, Im MDSC AC, and CD33 on CD66b^++^ myeloid cells, as well as protective factors such as CM CD8^+^ T cell % CD8^+^ T cell, HLA DR^+^ T-cell AC, CD25 on switched memory B cell, IgD on transitional B cell, CD16-CD56 on NKT, and SSC-A on HLA DR^+^ T cell.

In recent years, two main perspectives on the role of microbiota in this disease have emerged: direct infection of the central nervous system by microorganisms and indirect pathways involving the regulation of peripheral immune and metabolic systems (Seo and Holtzman, 2024). The peripheral immune system interacts closely with gut microbes (Fung et al., 2017), and blood-derived leukocytes have been observed in the brains of AD patients and animal models (Zenaro et al., 2015; Marsh et al., 2016). In mouse models with amyloid deposits, inhibition of Treg cells leads to a reduction in Aβ (Yeapuri et al., 2023). These studies support the idea that gut microbiota modulate peripheral immunity, affecting the occurrence of AD. Wang et al. found that gut dysbiosis promotes the infiltration of peripheral immune cells in a mouse model of amyloidosis (Wang et al., 2019), further validating this perspective. Therefore, we used a mediation Mendelian randomization approach to explore whether gut microbiota can influence the occurrence of AD by affecting immune cells. Excitingly, we discovered that genus *Oscillibacter* partly inhibits the occurrence of AD by promoting EM CD4^+^ T-cell AC. The remaining 16 immune cells could serve as potential mediators of the influence of 13 gut microbiota and related pathways on AD occurrence. Multiple immune cell types are influenced by various gut microbiota, such as CD39^+^ resting CD4^+^ Treg % CD4^+^ Treg being positively regulated by stearate biosynthesis II in bacteria and plants and negatively regulated by order Coriobacteriales. Similarly, CD4RA on TD CD4^+^ T cell is positively regulated by pantothenate and coenzyme A biosynthesis III and negatively regulated by molybdenum cofactor biosynthesis. These factors may contribute to the insignificance of gut microbiota influencing AD through immune cell modulation. Although the above results indicate that immune cells mediate the impact of gut microbiota on AD, the proportion of immune cell mediation is only 5–10%, explaining only part of the mechanism by which gut microbiota affect distant AD pathology. Therefore, more mediators need to be considered to comprehensively explain the impact of gut microbiota on AD.

## Conclusion

In this study, we comprehensively explored the causal effects among gut microbiota, immune cells, and AD. We identified causal relationships between 13 gut microbiota species and AD, with 17 immune cells mediating the effects to a certain extent. Among these, the most significant was genus *Oscillibacter* with EM CD4^+^ T-cell AC accounting for 8.99% of its inhibitory effect on AD occurrence. However, much of the impact of gut microbiota on AD remains unclear. Further research is needed to investigate other potential mediators of risk factors. In addition, the generalizability of our study results may be limited by the fact that the initial GWAS data primarily came from European populations. Future research should include larger and more diverse populations.

## Data availability statement

The original contributions presented in the study are included in the article/Supplementary material, further inquiries can be directed to the corresponding authors.

## Author contributions

HuaZ: Data curation, Writing – original draft. YW: Methodology, Writing – original draft. HuiZ: Funding acquisition, Supervision, Writing – review & editing, Investigation. WW: Data curation, Investigation, Writing – original draft. FH: Funding acquisition, Supervision, Writing – review & editing.

## Footnotes

^1^ https://gwas.mrcieu.ac.uk
